# Evaluating Messaging on Prenatal Health Behaviors Using Social Media Data: Systematic Review

**DOI:** 10.2196/44912

**Published:** 2023-12-20

**Authors:** Nessie Felicia Frennesson, Cheryl McQuire, Saher Aijaz Khan, Julie Barnett, Luisa Zuccolo

**Affiliations:** 1 Tobacco and Alcohol Research Group School of Psychological Science University of Bristol Bristol United Kingdom; 2 Centre for Public Health Bristol Medical School University of Bristol Bristol United Kingdom; 3 National Institute for Health and Care Research School for Public Health Research Newcastle United Kingdom; 4 Population Health Sciences Bristol Medical School University of Bristol Bristol United Kingdom; 5 Department of Psychology University of Bath Bath United Kingdom; 6 Health Data Science Centre Human Technopole Milan Italy; 7 Medical Research Council Integrative Epidemiology Unit University of Bristol Bristol United Kingdom

**Keywords:** acceptability, design, development, effectiveness, health behavior, health messaging, messaging, prenatal health, prenatal, social media data, social media, tool

## Abstract

**Background:**

Social media platforms are increasingly being used to disseminate messages about prenatal health. However, to date, we lack a systematic assessment of how to evaluate the impact of official prenatal health messaging and campaigns using social media data.

**Objective:**

This study aims to review both the published and gray literature on how official prenatal health messaging and campaigns have been evaluated to date in terms of impact, acceptability, effectiveness, and unintended consequences, using social media data.

**Methods:**

A total of 6 electronic databases were searched and supplemented with the hand-searching of reference lists. Both published and gray literature were eligible for review. Data were analyzed using content analysis for descriptive data and a thematic synthesis approach to summarize qualitative evidence. A quality appraisal tool, designed especially for use with social media data, was used to assess the quality of the included articles.

**Results:**

A total of 11 studies were eligible for the review. The results showed that the most common prenatal health behavior targeted was alcohol consumption, and Facebook was the most commonly used source of social media data. The majority (n=6) of articles used social media data for descriptive purposes only. The results also showed that there was a lack of evaluation of the effectiveness, acceptability, and unintended consequences of the prenatal health message or campaign.

**Conclusions:**

Social media is a widely used and potentially valuable resource for communicating and evaluating prenatal health messaging. However, this review suggests that there is a need to develop and adopt sound methodology on how to evaluate prenatal health messaging using social media data, for the benefit of future research and to inform public health practice.

## Introduction

### Background

Accurate and easily understandable information on prenatal health is important for maternal and child health. There are several poor health outcomes among newborns that are affected by prenatal health behaviors. For example, alcohol consumption during pregnancy can result in fetal alcohol spectrum disorders, leading to lifelong developmental disabilities and multiple comorbidities [1,2], and smoking during pregnancy can, among other outcomes, lead to low birthweight [3].

The internet is used by people all around the world. As many as 5.48 billion of the world’s population are reported to be using the internet [4]. In terms of social media use, Facebook alone had 2.4 billion users in 2019 [5]. Facebook is not the only social media platform that is widely used. In 2021, 71% of Americans aged between 18 and 29 years reported using Instagram, with 65% reporting using Snapchat [6]. Gender differences are also noticeable in social media usage. Men are said to use social media to gain information, while women are reported to use social media to keep up with relationships [7]. This shows the importance of ensuring that information targeting women online reaches the intended audience.

Moreover, there is a growing recognition that social media plays a key role in society today, not only being an important means of communication but at the same time being influential in health decisions [8]. Social media has been proven to spread information and create engagement quickly [9]. Social media can also be used by researchers and public health agencies to gain real-time insight into people’s attitudes and behaviors, as well as identify how people perceive public health messages [10]. It is, therefore, timely and important to investigate how and to what extent social media data have been used to evaluate prenatal health messaging. Zhu et al [11] reported that the majority of their participants, expecting mothers, had a good experience seeking information related to pregnancy on social media, and that had a positive impact on them. They reported, for example, that the participants could find support on social media and that it reduced anxiety and loneliness. Moreover, Baker and Yang [12] reported that 84% of their respondents, new mothers, thought of friends on social media as being supportive during pregnancy. Not only can social media be a source of information, but it can also help pregnant women feel more in control over their pregnancies and the health decisions they are making [13]. It has been shown that most pregnant women use the internet to search for information at least once a month during pregnancy [14]. Ford and Alwan [15] showed that around 20% of the pregnant women who responded to the survey used social media to find information on vaccinations during pregnancy. Other information that is searched for related to pregnancy is, for example, nutrition, medication, and antenatal care [14]. It is also important to note that there are concerns about searching for health-related information online. According to the World Health Organization (WHO) [16], an infodemic, meaning too much information or false information, could lead to worsening health outcomes. Misinformation about health is present on all different types of social media platforms [17].

With the recent COVID-19 pandemic, pregnant women were advised to minimize their in-clinic visits and instead meet with their health care providers over the phone or through video calls [18]. In the absence of in-person consultations, social media could have been the initial and only source of information pertaining to health and well-being; therefore, it can play a substantial role in influencing health behaviors during pregnancy.

Since social media is commonly used as a source of information, it is important that the information available on social media is both accepted by the intended audience and effective in changing the targeted health behavior. Moreover, it is important that the information reaches the audience that it intends to. Others have investigated how health messages delivered on social media have been evaluated [19]. However, this did not include a specific investigation of messages relating to prenatal health. Given the variability in the quality of information and the misinformation known to occur on social media, and related concerns that health messaging can potentially lead to increased anxiety among pregnant people, there is a need to review the impact, acceptability, effectiveness, and unintended consequences of prenatal health messaging and campaigns. Therefore, this systematic review aims to address this important gap in research by reviewing the current research on how prenatal health messaging and campaigns have been evaluated to date using social media data.

### Aims

The specific research questions in this systematic review were:

1. What research has been carried out on evaluating official messaging and campaigns on prenatal health behavior using social media data?
2. Which methods have been used to evaluate the impact of official messaging and campaigns on prenatal health behavior using social media data?
3. To what extent has previous research looked at the impact, effectiveness, acceptability, and unintended consequences of official prenatal health messaging and campaigns?
4. What is the methodological quality of previous research that has evaluated official messaging and campaigns related to prenatal health?

## Methods

### Search Strategy

This systematic review was registered with PROSPERO (International Prospective Register of Systematic Reviews; CRD42022315743). The search strategy involved multiple steps. First, search terms were developed by the review team based on keywords from the relevant literature, expert knowledge, and consultation with library service colleagues. The following words were identified as suitable for the search: “Pregnan*,” “Prenatal,” “F?etal,” “Antenatal,” “Gestation*,” “Matern*,” “Mother,” “Health,” “Behavi*,” “Evaluati*,” “Efficac*,” “Effective*,” “Messag*,” “Campaign,” “Communication,” “Media,” “Social Media,” “Social Network Sites.”

Searches were conducted in the following databases: PubMed (MEDLINE), PsycINFO, Web of Science, the Cochrane Library, and Scopus. The search string was adjusted depending on the database, together with relevant Medical Subject Headings terms and Boolean operators. The final search string for PubMed can be found in Multimedia Appendix 1. The search was carried out on March 23, 2022, with a final search conducted on June 14, 2022. Restrictions were made to only include articles in English or Swedish. Second, Google Scholar was searched using the same sets of search terms, screening the first 100 results and including the results if relevant. Third, manual searches of reference lists in the relevant literature were carried out. Final, Google Scholar was used to forward search for further relevant articles. When necessary, the article author or authors were contacted when full texts were not available.

### Inclusion and Exclusion Criteria

Studies were included if they fit the following criteria: (1) the study had to describe an official message or campaign (for example, from a recognized health organization) targeting prenatal health behaviors. Evaluation of peer advice was not included, (2) the study had to target pregnant women or the general public, (3) the study had to use social media data (eg, likes, comments, and shares) to evaluate the message or campaign, and (4) the study had to be available in English or Swedish. Online as well as offline messaging or campaigns were included in the review, as long as they were evaluated using social media data. Studies with interventions related to preconception behavior were excluded.

Both quantitative and qualitative studies were considered, and eligible studies included systematic reviews, randomized controlled trials, nonrandomized controlled studies, observational studies, natural experiments, and qualitative studies. Moreover, gray literature sources, such as reports, blogs, and conference proceedings, were considered.

### Overlapping Studies

A total of 2 included studies analyzed the same campaign [20,21]. After careful consideration among reviewers, the decision was made to keep them both in the review since they used different methods for analysis.

### Data Extraction and Analysis

The identified articles were assessed according to the inclusion criteria listed above. The first reviewer (NFF) screened the study titles and abstracts. A random selection of 10% (2/13) of the full texts was assessed by the third reviewer (SAK) for eligibility as agreed on in the review protocol. Reasons for exclusion were documented throughout the full text screening process. There was no discrepancy, and consensus was reached between the reviewers, so there was no need for consultation by the other reviewers at this stage.

The first reviewer (NFF) independently extracted the data agreed on in the preregistered review protocol (PROSPERO CRD42022315743), including sample population characteristics (eg, geographical location) and message or campaign characteristics (eg, message or campaign topic, objectives of the message or campaign, and message or campaign delivery). The full list of the data extracted can be found in Multimedia Appendix 2. The third reviewer (SAK) repeated the data extraction for 10% of the articles to ensure accuracy. In the case of full texts missing or when clarification was needed, the article author or authors were contacted. The studies were summarized narratively.

Articles described and measured outcomes differently. However, the following definitions were applied to all articles: Effectiveness was defined as a change of attitudes, behavior, or knowledge relating to the behavior targeted. Acceptability was defined as either qualitatively to what level the campaign or message was agreed with or rejection of the campaign or message, or quantitatively as the click-through rate as previously used in social media data analysis [22]. Unintended consequences were defined as any consequence or effect that was not intended for the messaging or campaign (eg, stigmatizing certain behaviors or individuals). Impact was defined as each paper’s social media metrics (eg, likes and shares). Only outcomes measured using social media data were evaluated in this systematic review.

A content analysis with a thematic synthesis approach was used to summarize qualitative evidence. Quantitative summary statistics were used when applicable. No subgroup analysis could be performed as there were not a sufficient number of studies identified.

### Quality Assessment of the Studies

Despite the increasing number of studies using social media data, no relevant validated quality assessment tool could be identified. There are several important factors to take into account when assessing the quality of social media data that have been discussed by several scholars [23-25]. Therefore, after considering previous discussions around social media data quality, we make use of the assessment tool developed by Golder et al [26] and further refine it to the following criteria:

1. Bias: for example, if applicable, has sampling bias been accounted for? If intervention or campaign, has it been reported whether the intended population has been reached?
2. Choice of data: for example, can social media data help answer the study objectives? How well has the selection of specific social media channels been argued for?
3. Data extraction: for example, are the methods that have been used to extract the social media data reported? If there is an extraction of comments or tweets, has there been a double extraction of the data?
4. Statistical analysis: for example, is the analysis method appropriate for the objective of the study?

The studies were rated as either low quality, high quality, or cannot tell. The studies were assessed based on their social media data and methodology; no other eventual data source or methodology was used. The first reviewer (NFF) assessed all included studies, with the third reviewer (SAK) assessing 3 of the studies. Any disagreement was discussed between the reviewers.

## Results

### Result of the Search

**Figure 1:**
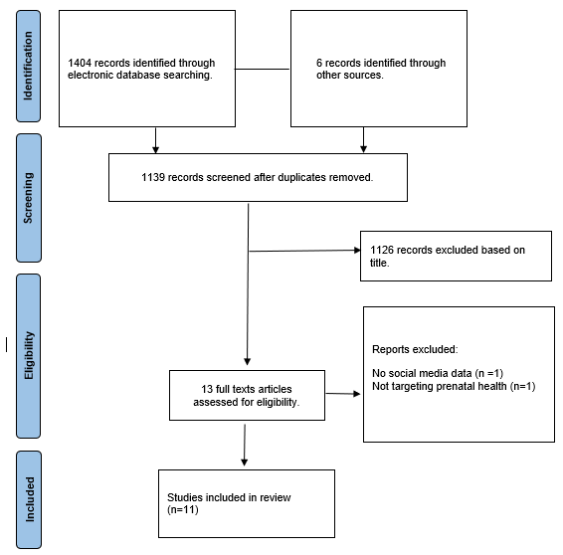
PRISMA (Preferred Reporting Items for Systematic Review and Meta-Analysis) flowchart depicting the selection of studies.

The initial screening identified 1404 records, with another 6 identified by searching the first 100 Google Scholar results, as well as with backward and forward reference searches. This process resulted in a total of 11 articles meeting all inclusion criteria for this review. Figure 1 illustrates a flow diagram of the selection process. After full text screening, 1 article was excluded due to not targeting prenatal health specifically [27], and another article was excluded due to not using social media data [28].

### Characteristics of Included Studies

A total of 3 eligible studies targeted alcohol consumption during pregnancy [20,21,29], and 2 of the studies targeted maternal vaccination [30,31]. Another 2 studies were on the topics of weight gain, nutrition, and the importance of seeking prenatal care [32,33]. The remaining studies targeted seeking prenatal care if experiencing decreased fetal movements [34], healthy weight gain during pregnancy [35], maternal smoking [36], and nutrition during pregnancy [37]. All the included articles were published between 2016 and 2022. A majority (n=8) of the articles aimed to describe or evaluate campaigns and messaging [29,30,32-37], with the others aiming to understand the discourse around maternal vaccination [31], to understand how Facebook advertisements can be used to communicate public health messages [20], and to investigate the suitability of the dynamic transactional model to communicate public health [21].

Further characteristics of the included studies are shown in Table 1.

**Table 1:** Characteristics of included studies.

Study, year	Health behavior	Aim of the paper	Campaign or messaging population	Geographical location
Bazzo et al [29], 2016	Alcohol consumption during pregnancy	Describe a campaign intended to raise awareness about FASD^a^	General population	Worldwide
Bonnevie et al [33], 2021a	Weight gain, nutrition, and seeking prenatal care	Describe the campaign “Growing and Glowing” and its effectiveness	Black pregnant women	United States^b^
Bonnevie et al [32], 2021b	Weight gain, nutrition, and seeking prenatal care	Describe the campaign “Strong Beautiful Future” and its feasibility	Black pregnant women	United States^c^
Carlson et al [30], 2019	Maternal influenza vaccination	Evaluate a campaign intended to increase awareness of maternal influenza vaccination and determine sources of information and attitudes	Pregnant women	Australia
Chan et al [34], 2021	Seeking prenatal care if experiencing decreased fetal movements	Describe the campaign “Movement Matter” and its effectiveness	Women	Australia
Graham et al [35], 2019	Healthy weight gain during pregnancy	Describe the implementation of a campaign intended to promote healthy weight gain during pregnancy	Women	Not country specific
Martin et al [31], 2020	Maternal vaccination	Understand the discourse around maternal vaccination on social media	Pregnant women	15 countries^d^
Miller et al [36], 2022	Smoking during pregnancy	Evaluate dissemination efficacy and examine reach and engagement with a Facebook campaign about risks of smoking during pregnancy	Pregnant women	United States^e^
Parackal et al [20], 2017	Alcohol consumption during pregnancy	Understand the communication process of Facebook advertisement communicating public health messages	Women	New Zealand
Parackal et al [21], 2021	Alcohol consumption during pregnancy	Investigate the suitability of the Dynamic Transactional Model to communicate public health messages	Women	New Zealand
Verduci et al [37], 2021	Nutrition during pregnancy and early life	Describe the introduction of a new tool of eHealth communication to communicate health messages	General population	Italy

^a^FASD: fetal alcohol spectrum disorders.

^b^Hillsborough County, Florida.

^c^Orange County, Florida.

^d^Australia, Brazil, Canada, France, Germany, India, Italy, Mexico, Panama, South Africa, South Korea, Spain, Taiwan, the United Kingdom, and the United States.

^e^New Jersey, Massachusetts, Georgia, North Carolina, Louisiana, and Kentucky.

### Methodology of Included Studies

Table 2 presents the included studies’ methodology. Each of the included articles used data obtained from social media channels. A wide range of metrics were used to evaluate the messaging and campaigns, making comparisons between them difficult. Around 6 of the articles used social media data mainly to present social media metrics and metadata. A majority of the studies (n=7) [29-34,37] used more than 1 social media channel as a source of data. The following social media channels were used:

- Facebook (n=11) [20,21,29-37].
- Twitter (subsequently rebranded X; n=6) [29,31-34,37].
- Instagram (n=6) [30-34,37].
- YouTube (n=1) [32].

**Table 2:** Methodology and data source of included studies.

Study	Social media channel	Social media data analysis	Additional data source used in paper	Survey population
Bazzo et al [29], 2016	Facebook and Twitter	Social media metrics^a^	Survey	Survey to the partner organizations that distributed the campaign
Bonnevie et al [33], 2021a	Instagram, Facebook, and Twitter	Social media metrics	Survey and website data	Women, aged 18-65 years living in Hillsborough Country Florida, United States
Bonnevie et al [32], 2021b	Instagram, Facebook, Twitter, and YouTube	Social media metrics	Survey and website data	Women, aged 18-65 years, living in Orange County Florida, United States^b^
Carlson et al [30], 2019	Instagram and Facebook	Social media metrics	News articles and survey	English speaking pregnant women, aged 18 years or older attending an antenatal clinic in western Sydney, Australia
Chan et al [34], 2021	Instagram, Facebook, and Twitter	Social media metrics	Survey	Clinicians working from one of the designated clinics in Victoria, Australia. Pregnant women at ≥28 weeks gestation receiving care at one of the same clinics
Graham et al [35], 2019	Facebook	Social media metrics	N/A^c^	N/A
Martin et al [31], 2020	Instagram, Twitter, blogs, and forums	Social media metrics, discourse analysis, topic analysis, and stance analysis	None	N/A
Miller et al [36], 2022	Facebook	Social media metrics and content analysis	None	N/A
Parackal et al [20], 2017	Facebook	Social media metrics, thematic analysis, logistic regression, and sentiment analysis	None	N/A
Parackal et al [21], 2021	Facebook	Cluster analysis and regression analysis	None	N/A
Verduci et al [37], 2021	Instagram, Facebook, and Twitter	Social media metrics	Website and app data	N/A

^a^Social media metrics includes number of impressions, users, interactions, likes, tweets, reach, engagement, views, clicks, followers, reactions, and shares.

^b^Only Black women were included in the analysis.

^c^N/A: not applicable.

A total of 2 articles reported studies that used paid influencers to further spread their message and campaigns on social media platforms [32,33]. Martin et al [31] extracted social media data to analyze the discourse around maternal vaccination using stance, discourse, and topic analysis. Miller et al [36] conducted a content analysis. Parackal et al [20] performed a cluster analysis by using text mining techniques on comments extracted from a campaign against drinking alcohol during pregnancy as well as performing a logistic regression to find relationships between meaning-making themes and the message to abstain from alcohol. Parackal et al [21] used the same campaign to investigate the suitability of the dynamic transaction model when using social media to communicate health messages.

### Effectiveness, Acceptability, Impact, and Unintended Consequences

Table 3 presents the study’s main results in terms of effectiveness, acceptability, impact, and unintended consequences.

**Table 3:** Summary of effectiveness, acceptability, impact, and unintended consequences of included studies.

Study	Measurement of effectiveness	Measurement of acceptability	Measurement of impact	Measurement of unintended consequences
Bazzo et al [29], 2016	Not measured	Not measured	Impressions^a^, users^b^, interactions^b^, likes, and tweets during launch week	Not measured
Bonnevie et al [33], 2021a	Not measured	Not measured	Average monthly impressions^a^, average daily reach (number of people who have seen the content), average monthly engagement (number of likes, comments, shares, video views, and clicks on posts)	Not measured
Bonnevie et al [32], 2021b	Not measured	Not measured	Average monthly impressions^a^, average daily reach (number of unique people who have seen the content), average monthly engagement (number of likes, comments, shares, video views, and clicks on posts)	Not measured
Carlson et al [30], 2019	Not measured	Click-through rate from Facebook to the NSW^c^ Health website	Average monthly impressions^a^, average daily reach (number of unique people who have seen the content), average monthly engagement (number of likes, comments, shares, video views, and clicks on posts)	Not measured
Chan et al [34], 2021	Not measured	Not measured	Impressions^a^, estimated combined reach^b.^, likes, and followers	Not measured
Graham et al [35], 2019	Not measured	Click-through rate on Google Ads to HPHC^d^ website and click-through rate on Facebook ads to HPHC website	Impressions^a^, reactions, shares, and comments	Not measured
Martin et al [31], 2020	Not measured	Stance analysis	Not measured	Semantic network, topic analysis, and stance analysis
Miller et al [36], 2022	Not measured	Content analysis	Impressions^a^, reach (Number of unique individuals), likes, reactions, shares, comments, and video views	Not measured
Parackal et al [20], 2017	Not measured	Thematic analysis	Likes, comments, shares, and views	Not measured
Parackal et al [21], 2021	Not measured	Not measured	Not measured	Not measured
Verduci et al [37], 2021	Not measured	Parents’ comments on social media channels and blogs	Views, reach^b.^, interactions^b^, likes, and shares	Not measured

^a^Facebook impressions are measured as to how often the content is displayed to the audience [38].

^b^Not specified how it was measured.

^c^NSW: New South Wales.

^d^HPHC: Healthy Parents, Healthy Children.

#### Effectiveness

None of the included studies measured the effectiveness of the messaging or campaign.

#### Acceptability

A total of 6 studies provided data on acceptability, which was heterogeneously defined. A total of 2 studies used click-through rates (defined as the number of clicks per the number of impressions) as a measure of acceptability, showing rates ranging from 0.4% to 5.8% [30,35].

A total of 3 more studies used a variety of other methods for assessing acceptability, including thematic analysis and stance analysis. In their study of maternal vaccination discourse, Martin et al [31] showed that the messaging of around 42% of the tweets could be classified as having a promotional stance toward maternal vaccination, with the rest being either neutral, ambiguous, or discouraging in their stance. Miller et al [36] in their study about informing women about the risks of maternal smoking reported more comments showing skepticism or disbelief (n=44) toward the message than comments showing support or belief (n=25), some of which said that the message was not believed or that there was no proof for it. By looking at comments posted on their social media platforms and blogs, Verduci et al [37] claimed that the website was helpful in increasing knowledge and awareness of the importance of nutrition during pregnancy. Parackal et al [20] noted the low acceptability of the campaign targeting alcohol consumption during pregnancy, with typical comments including words such as “stupid.”

#### Impact

All but 2 of the included studies presented social media metrics such as impressions, engagement, views, users, followers, interactions, likes, comments, shares, tweets, and reach as the measure of impact.

Chan et al [34] reported both the actual reach of the Facebook campaign (620,536 women) and the 85% percentage of the target audience on Facebook that it represented.

Not all studies provided the same metrics, making any direct comparison between campaigns impossible. However, the highest number of impressions (115,450 on Facebook) was provided by Miller et al [36], the highest number of views (201,754 on Facebook videos) was provided by Parackal et al [20], and the highest number of likes (19,600 likes on the Facebook page) was provided by Verduci et al [37].

#### Unintended Consequences

Although not the main focus, one study presented data on the unintended consequences of health messaging. Martin et al [31] identified negative conversations and discouraging tweets toward maternal vaccination, claiming links to autism and fetal deaths, as well as a mistrust toward public health authorities.

#### Quality Assessment

Within the 3 studies [21,32,35] that were double assessed, 2 ratings were disagreed on. However, after discussion, the reviewers agreed on the final rating. Moreover, it was also agreed that no studies should be excluded.

**Figure 2:**
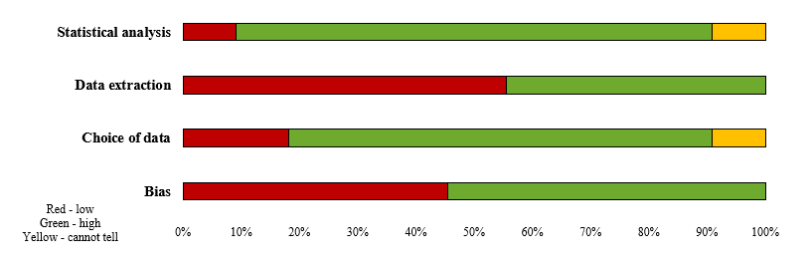
Methodological quality ratings for each criterion across all included studies.

It was noted that the biggest concern was that many of the included studies did not report how the data had been extracted, as well as whether the intended audience had been reached by the message or campaign. Figure 2 presents the methodological quality of the included studies.

## Discussion

### Principal Results

The purpose of this systematic review was to retrieve and analyze previous research on evaluating the impact, effectiveness, acceptability, and unintended consequences of messaging on prenatal health behaviors using social media data. The secondary aim was to review the methods that had been used to carry out these evaluations, both in terms of methodology and the quality of the studies. Despite the number of articles identified in the first screening process (n=1139 after duplicates were removed), this review suggests that not many studies have used data from social media to evaluate prenatal health messaging and campaigns. This was unexpected since social media plays a key role in providing health information to the public [8].

This review highlighted a gap in the literature in terms of evaluating the effectiveness, acceptability, and unintended consequences of prenatal health messaging. The majority of studies reported impact in terms of social media metrics (eg, impressions, reach, shares, and comments), while a study [34] presented the actual impact (reach) of the campaign in terms of the size of the audience that they reached on Facebook. None of the included studies evaluated the effectiveness of the message or campaign by using social media data. For future research, it is important to create a reliable methodology to measure the effectiveness of prenatal health messaging or campaign. It has been noted that there is no consensus on how effectiveness is defined in this context, together with the issue of the feasibility of assessing effectiveness due to the lack of follow-up in many studies that use social media data. Other studies have, for example, evaluated effectiveness by using sales numbers for condoms after a social media campaign promoting condom use in Turkey [39]. Thus, many studies rely on the availability of other data sources than social media for effectiveness evaluation. This is also true for measuring the acceptability of the messaging or campaign. A total of 2 included studies [20,36] showed comments expressing a negative stance or skepticism, for example, the message was not believed or accused of not being evidence-based. A way of evaluating the acceptability of prenatal health messaging or campaigns could be to do a pilot launch of the intended information and only release it to a limited audience, and thereafter see how well the messaging is received. A total of 3 included studies had external people reviewing the content before the campaigns were launched [29,33,36]. However, none of them used social media data to evaluate this. Therefore, this information was not included in this review. Particularly neglected is the area of unintended consequences, with reports from only one study. Monitoring unintended consequences both in terms of broader prenatal health messaging as well as during the rollout of a communication campaign is a crucial course correction step as it can allow improvisation of health messages (if done prospectively and in a timely fashion) or at least to accumulate “lessons learnt” for a more realistic and rounded evaluation of the campaign’s success (when done retrospectively).

An important factor when communicating and evaluating prenatal health messaging and campaigns is to recognize that the type of information sought can vary depending on whether it is on the internet or on social media. This includes search engines being used to search for health-related information and social media being used to find out more about the impact of health conditions [40]. Moreover, in a study conducted by Daly et al [41], 99.5% of the respondents found health websites and apps an acceptable place for health information, while 88.2% agreed that social media was acceptable.

A total of 2 articles in this systematic review used influencers to spread their campaign [32,33]. It has been shown that there is a risk of misinformation on social media, especially health information spread by influencers [42]. This creates an online environment that needs accurate prenatal health information that reaches the intended audience, again showing the importance of assessing that the information is reaching the intended audience and is also accepted by them. Moreover, if using influencers to spread prenatal health messaging or campaigns, it is of priority to ensure that the influencers are disclosing if they are being paid to create trustworthiness for the information.

The studies in this review varied in how they used social media data, with most of them using it for descriptive purposes. Other than that, there was no consistent methodology among the included studies. This aligns with a previous systematic review looking at health promotion interventions using social media networks, showing that a wide range of measures are used to evaluate social media health messaging relating to a wide range of topics [19]. Moreover, the methodological quality of the studies was assessed using a quality assessment tool adapted from Golder et al [26]. The biggest concern for the methodological quality was the choice of social media platform, especially the lack of a priori justification behind the selection of the type of social media data. This is crucial as audience characteristics may vary between social media platforms; as a consequence, the selection of a specific social media platform may have implications for health messaging. Scholars have argued that we live in a society of digital inequality where those who engage in the digital world have a greater advantage than those who do not [43]. Moreover, it has been shown that higher socioeconomic status has a relationship with a broader use of social media platforms and other digital media platforms [44]. The use of the internet and social media also differs between countries. In some low- and middle-income countries, less than 5% of the population is online [45]. Again, this highlights the importance of having the correct methodology to measure whether the intended audience is reached or if they can even be reached by the information.

In addition to the uptake of health messaging, there are notable variations between the measurements of impact or impact metrics across the social media platforms (eg, retweets on Twitter or reach on Facebook). For future research, we suggest strengthening evaluations of messaging and campaigns related to prenatal health behaviors to create a robust methodology and trustworthy results, for example, by using causal inference methods applied to social media data and providing clear definitions of outcomes of interest, for example, effectiveness, acceptability, and were unintended consequences.

### Strengths and Limitations of This Review

The principal strength of this review is its comprehensive and systematic approach. This extends to the inclusion of diverse social media platforms and targeting studies using any kind of social media data in a broad sense, even if the campaign or message was not communicated on social media channels. Moreover, the search included studies that did not have a main aim analyzing social media data but were still considered eligible if social media data were used. Second, the definitions of effectiveness, acceptability, and unintended consequences used in this review were not based on how the included articles had defined them since these definitions were often not provided. Instead, the definitions were decided upfront and applied to all studies to ensure consistency. Nevertheless, there are limitations of this review, such as language limitations. Given the spoken languages of the reviewers, only English and Swedish articles were eligible. Finally, to minimize publication bias, gray literature was included in the search strategy. Regardless of the measures to reduce publication bias, it must be noted that studies reporting significant positive results are more likely to be published than those reporting negative or no impact [46].

### Comparison With Previous Work

This is the first review of the evaluation of prenatal health messaging using social media data. Previous systematic reviews have evaluated interventions delivered on social media, targeting any health behavior [19] as well as evaluating digital interventions [47]. Lim et al [19], in accordance with the evidence in this review, showed a lack of consistent and robust methodology among the included studies when it came to evaluating the effectiveness of health promotion interventions delivered on social media. Unlike this review, Lim et al [19] included studies not using social media as a data source for analysis, which allowed the inclusion of more studies (n=47). Another systematic review looking at health behaviors and social media is the one conducted by Chang et al [48] which looked at social media and weight management. Similar to this review, they reported a need for future research to measure social media’s role and effectiveness in influencing health behaviors.

### Conclusions

Social media could be a valuable resource, both for communicating and evaluating prenatal health messaging and campaigns. This is true for those who are interested in capturing real-time data about any health care messaging through social media around a variety of health care domains. Previous research on evaluating prenatal health messaging using social media data showed that there is a need to develop a universal understanding of what measurements to use to carry out these evaluations. This is especially true with regard to measuring the effectiveness of the messaging, making sure that the message is clearly understood, and shaping behaviors. By developing a comprehensive set of recommendations covering all the evaluation steps, there is an exciting possibility for future research to be able to contribute to a better understanding of prenatal health messaging by using social media data as the rich data source it is.

## Appendix

Multimedia Appendix 1 Search strategy.

Multimedia Appendix 2 Full list of data extracted.

Multimedia Appendix 3 PRISMA checklist.

## Abbreviations

FASD: fetal alcohol spectrum disorders
HPHC: Healthy Parents, Healthy Children
NSW: New South Wales
PROSPERO: International Prospective Register of Systematic Reviews
WHO: World Health Organization
