# Supplementary material for: Evaluating Messaging on Prenatal Health Behaviors Using Social Media Data: Systematic Review
Source: J Med Internet Res. 2023 Dec 20;25:e44912. doi: 10.2196/44912 (PMC10765287; doi:10.2196/44912)
Supplement: Multimedia Appendix 1 [file jmir_v25i1e44912_app1.docx]

**APPENDIX 1:**

**PubMed search string:**

Search: ((("Pregnan*") or ("Pregnancy"[Mesh])) OR ("prenatal") or ("prenatal care") or ("Prenatal Care"[Mesh]) OR ("prenatal education") or ("Prenatal Education"[Mesh])) OR ("Fetal") or ("foetal") OR (("fetus") ("foetus") or ("fetus"[MeSH Terms])) OR ("antenatal") OR ("Gestation*") OR ("Matern*") OR (("mother") or ("Mothers"[Mesh]))) AND (("health") or ("Health"[Mesh])) AND (("behavi*") or ("Behavior"[Mesh]) OR ("attitude*") or ("Attitude"[Mesh])) AND (("Evaluati*") OR ("Effic*" ) OR ("effective*")) AND (("media" ) OR ("Social network site*") or ("social media") or ("Social Media"[Mesh])) AND (("messag*") OR ("campaign") OR ("communication") or ("Communication"[Mesh]) OR ("health promotion") or ("Health Promotion"[Mesh])) Filters: English, Swedish
