# Supplementary material for: Evaluating Messaging on Prenatal Health Behaviors Using Social Media Data: Systematic Review
Source: J Med Internet Res. 2023 Dec 20;25:e44912. doi: 10.2196/44912 (PMC10765287; doi:10.2196/44912)
Supplement: Multimedia Appendix 2 [file jmir_v25i1e44912_app2.docx]

Extracted data

Name of article

Authors

DOI/URL

Journal/Source

Article funding

Reported conflict of interest

Contact details to author

Publication type

Year of publication

Targeted population

Age

Ethnicity

Gender

Geographical location of message/campaign

Comparison group (Y/N)

Targeted health behaviour

Definition of health behaviour

Theory used (Y/N)

Method of analysis (of social media data)

Software used for data extraction

Social media channel used

Message/campaign topic

Objective of message/campaign

Hashtags

Date of campaign/message

Duration of message/campaign

Message/campaign delivery

Evaluation of effectiveness

Evaluation of acceptability

Evaluation of unintended consequences

Social media data (Instagram, Facebook, Twitter, YouTube, Google Ads)

Likes

Followers

Impressions

Reactions

Tweets

Reach

Click-Throughs

Engagement

Views

Analysis methods
